# The Non-Activated Thromboelastometry (NATEM) Assay’s Application among Adults and Neonatal/Pediatric Population: A Systematic Review

**DOI:** 10.3390/diagnostics12030658

**Published:** 2022-03-08

**Authors:** Petroula Georgiadou, Rozeta Sokou, Andreas G. Tsantes, Stavroula Parastatidou, Aikaterini Konstantinidi, Dimitra Houhoula, Styliani Kokoris, Nicoletta Iacovidou, Argirios E. Tsantes

**Affiliations:** 1 Neonatal Intensive Care Unit, “Agios Panteleimon” General Hospital of Nikea, 18454 Piraeus, Greece; petroulag@yahoo.gr (P.G.); stavroula.parastatidou@gmail.com (S.P.); kmaronia@gmail.com (A.K.); 2 Laboratory of Haematology and Blood Bank Unit, “Attiko” Hospital, School of Medicine, National and Kapodistrian University of Athens, 12462 Athens, Greece; andreas.tsantes@yahoo.com (A.G.T.); dhouhoula@uniwa.gr (D.H.); stelkok19@gmail.com (S.K.); atsantes@yahoo.com (A.E.T.); 3 Neonatal Department, Medical School, Aretaieio Hospital, National and Kapodistrian University of Athens, 11528 Athens, Greece; niciac58@gmail.com

**Keywords:** thromboelastometry, non-activated thromboelastometry, NATEM, ROTEM, hemostasis, neonates

## Abstract

The non-activated thromboelastometry (NATEM) assay is a point-of-care assay that can provide a comprehensive insight into the actual hemostatic mechanism. However, there are very limited data about its use in clinical practice. The aim of this study was to systematically review the literature for any data regarding the use of NATEM in several clinical settings. A systematic review of PubMed and Scopus databases was conducted through 20 January 2022 for studies evaluating the use of the NATEM assay in different clinical settings. The literature search yielded a total of 47 publications, 30 of which met the eligibility criteria for this review. Evaluation of NATEM’s detecting ability for hemostasis disorders is limited in the literature. The results of the included studies indicate that NATEM seems to be a sensitive method for the detection of hyperfibrinolysis and may have an advantage in the diagnosis of hemostatic disorders. It could be more informative than the other ROTEM assays for detecting changes in coagulation parameters in patients who receive anticoagulants. However, the reported outcomes are highly varying among the included studies. NATEM has a high sensitivity to detect hypo- or hypercoagulability and provides a detailed insight into the whole hemostatic process from clot formation to clot breakdown. It could be a useful technique in variable fields of medicine, not only in adults, but also in pediatric and neonatal populations, to guide different hemostatic treatments and predict coagulation disorders or mortality/morbidity; this issue remains to be further investigated.

## 1. Introduction

Rotational thromboelastometry (ROTEM) is a point-of-care monitoring tool that provides a comprehensive overview of the coagulation process. It is used nowadays in many hospitals worldwide, while over the past years there has been a great interest in its efficacy to guide hemostatic therapy in various clinical settings. Viscoelastic tests such as thromboelastography (TEG) and ROTEM can detect changes in the viscosity and elasticity of whole blood samples during coagulation, from clot formation until clot breakdown, in order to provide diagnostic or prognostic information about any thrombotic or bleeding disorder [1,2,3].

ROTEM was the second viscoelastic test that was developed following thromboelastography. This assay allows the dynamic visualization of the viscoelastic properties of blood through a graphical representation of thrombin formation, fibrin polymerization and clot lysis, while it takes into account the complex interactions among different cellular populations, providing a point of care and a detailed evaluation of the patient’s hemostatic profile [4]. Existing evidence supports the utility of ROTEM assays in the diagnosis of bleeding conditions and in the prediction of mortality in critically ill patients as well as in predicting and guiding transfusion therapy [4,5,6,7,8]; the non-activated thromboelastometry (NATEM) assay is a particular test of ROTEM and evaluates both the intrinsic and the extrinsic coagulation pathways without the addition of any reagents for activation of coagulation. Hence, compared to the EXTEM and INTEM assays where tissue factor and ellagic acid are used, respectively; in order to activate coagulation, a NATEM assay may more accurately depict the hemostatic mechanism in vivo [4]. The NATEM assay evaluates the endogenous activation of hemostasis with a high sensitivity to detect any hyper- or hypocoagulation activity in the tested blood sample [3,4,9,10,11]. Therefore, NATEM could be a useful method to detect any changes in the hemostatic mechanism caused by multiple injuries, or in patients with diffuse intravascular coagulation (DIC). However, our knowledge regarding the role of the NATEM assay in the detection of hemostatic disorders is limited, especially in certain populations such as neonates and children.

We conducted a systematic review of the literature aiming to map the NATEM assay’s applications in various fields of medicine and to assess its role in detecting or predicting hemostasis disorders, or to guide hemostatic treatment. Another goal was to define the evidence-based reference range for the NATEM assay to diagnose hemostasis disorders in critically ill patients, especially in neonates with diseases, and to provide the sensitivity and specificity of its parameters.

## 2. Methods—Material

In order to perform this systematic review, a stepwise approach specified in the Preferred Reporting Items for Systematic Reviews and Meta-Analyses guidelines (PRISMA) was used [12].

### 2.1. Search Strategy

We systematically searched PubMed and Scopus databases through 20 January 2022. The keywords utilized were “Thromboelastometry”, “non- activated Thromboelastometry”, “NATEM”, “ROTEM”, in combination with Boolean logical operators (AND, OR). In addition, a manual electronic search of the references of the selected studies and of previous relevant systematic reviews was performed in order to fully cover the literature and to minimize the risk of missing a study.

### 2.2. Selection Criteria

We included all studies in English, evaluating the use of the NATEM assay in adults (patients or healthy volunteers) or in neonatal/pediatric populations.

### 2.3. Data Collection and Analysis

Reviewers were not blinded to the author or publication source of studies. The collected data for each study included the author, study design, year of the study, duration of the study, country, population characteristics, sample size, study objective and outcomes. We also retrieved information regarding the accuracy of NATEM parameters (sensitivity and specificity) to diagnose coagulopathies, to predict hemostasis disorders or mortality rate, and its use to guide hemostatic treatment. After duplicates were removed using the default settings in EndNote X7, the screening of titles and abstracts was performed by two team members (P.G. and R.S.). A.G.T and P.G. independently reviewed the full text of eligible studies in depth. Discrepancies in data extraction were resolved through a consensus and a third author had the decisive vote to resolve the disagreements (R.S). Furthermore, a manual search for any additional relevant articles was performed by two team members (S.P. and A.K). Due to the extreme methodological heterogeneity (in populations, methods/interventions and conditions being studied) of eligible studies, meta-analysis was deemed not feasible.

## 3. Results

**Figure 1:**
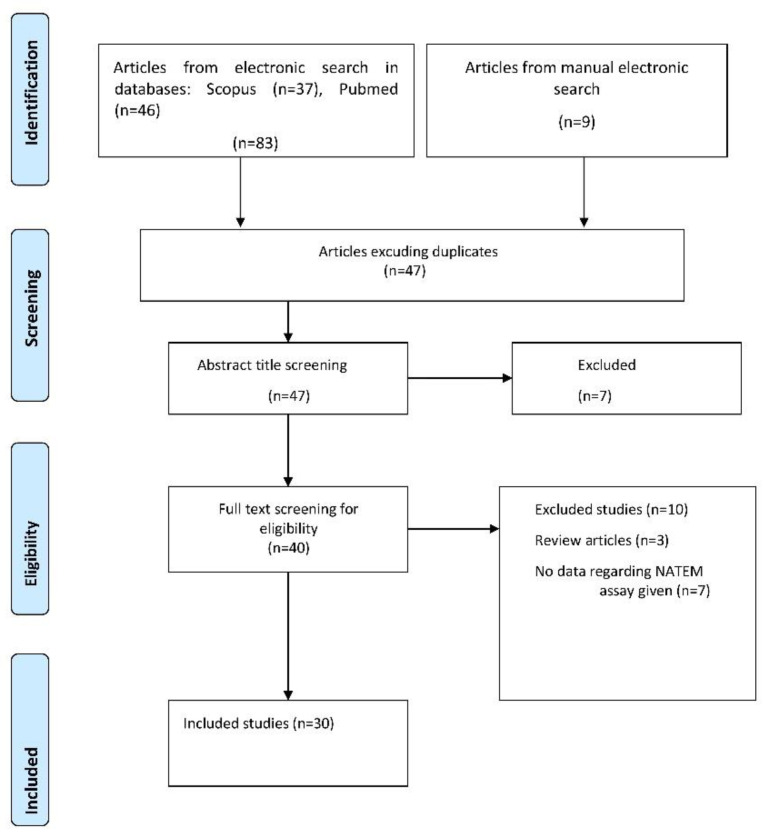
Flowchart of the literature review process.

After the removal of duplicates, our initial screen identified forty-six eligible studies in total. Seventeen studies were excluded after further in-depth reviewing of the studies because they did not include the evaluation of the NATEM assay, or they were not related to the aim of our search. The flowchart showing the search and study selection strategy is presented in Figure 1.

### 3.1. Study Characteristics

A total of 30 NATEM articles (1477 participants) from 2010 to 2022 were included. Twenty studies were conducted in Europe [13,14,15,16,17,18,19,20,21,22,23,24,25,26,27,28,29,30,31,32], three studies were conducted in Japan [33,34,35] and the USA [36,37,38], one study in Australia [39], one in Russia [40], one in Brazil [41] and one study was conducted in Israel [42]. The outcomes from the 30 included studies were grouped into four categories: experimental study using NATEM assay (*n* = 7), evaluation of NATEM assay in healthy human volunteers (*n* = 8), in patients versus healthy volunteers (*n* = 7) and in patients only (*n* = 8). All studies involved adults except from two studies that evaluated the NATEM assay in the neonatal population [32,42]. The median age of patients that was included in the studies ranged from neonatal age—three days of life—to 66 ± 15 years. All studies provided information related to the NATEM method in various fields of medicine.

Among the 30 studies included in the review, 23 were observational studies (including eight cohort studies, one case–control study and two case reports) whereas two were pilot studies and seven were in vitro experimental studies. There were no randomized control trials identified in our study. Seven studies enrolled a generally healthy population, while in two of them, the NATEM parameters were compared between animal (baboons or monkeys) and human populations [15,24]. Two studies assessed the impact of endothelial cells and in vitro albumin-induced dilutional coagulopathy on thromboelastometry parameters using the NATEM assay [16,18]. Two studies investigating the effects of naturopathic medicines on ROTEM and the effects of detergent sclerosants on clot formation and fibrinolysis, using ROTEM assays, were included in our study [23,39]. Seventeen studies evaluated the NATEM assay in various patient groups with different diseases, such as hemophilia A, cirrhosis with or without portal vein thrombosis, factor XI or VII deficiency, multiple myeloma, acute respiratory distress syndrome (ARDS), unclassified bleeding disorders, brain tumors, and disorders during pregnancy [14,17,19,20,21,22,26,27,33,34,35,37,40]. Only two studies enrolled neonates and the assessment of hemostatic profile and fibrinolysis in cord blood using NATEM [32,42].

### 3.2. Outcomes

#### 3.2.1. NATEM Assay Application in Experimental Studies

Seven studies focused on the NATEM assay application in experimental research (Table 1).

Bar et al. [37] performed a prospective experimental pilot study in order to assess coagulation by a NATEM assay in eight patients who received Rivaroxaban anticoagulant treatment, using chitosan or kaolin-based hemostatic agents. They revealed that the NATEM test could detect changes in coagulation parameters induced by hemostatic agents in patients receiving Rivaroxaban; generally, the study suggests that chitosan or kaolin-based hemostatic agents may be effective in improving these patients’ hemostasis profile. Bagge et al. [23] studied the effect of naturopathic medicines on hemostasis and demonstrated that the recommended standard intake of 1260 mg Ω-3 of polyunsaturated fatty acids (fish oil) daily may decrease platelet aggregation and clot formation. On the other hand, Parsi et al. [39] planned to investigate the in vitro effect of detergent sclerosants sodium tetradecyl sulphate (STS) and polidocanol (POL) on clot formation and fibrinolysis using the NATEM assay. Detergent sclerosants demonstrated a trimodal reaction on clot formation, initiating strong clot formation at low concentrations, weak clot formation at midrange concentrations and preventing clot formation at higher concentrations. Neither of the studied agents had fibrinolytic activity. Zipperle et al. [16] reported that adherent endothelial cells play a significant role in hemostasis and fibrinolysis and the evaluation of their contribution could be incorporated into a ROTEM assay by using coated microbeads. In a NATEM assay, only a significant reduction in clotting time (CT) value was observed in the presence of unstimulated endothelial cells, while on the other hand, clot formation time (CFT), a-angle, amplitude at 30 min (A30) and maximum clot firmness (MCF) values did not show any difference. Elvstam et al. [18] studied the ability of ROTEM to evaluate the effect of a four-factor Prothrombin complex concentrate (PCC) on an albumin-induced dilutional coagulopathy, and the effect of protamine to block the heparin activity. The authors concluded that since the NATEM assay is the most sensitive assay for detecting endogenous tissue factors, it may be the preferable method for monitoring the effect of PCC on dilutional coagulopathy. In another study by Scharbert et al. [29], the authors aimed to evaluate the heparin effect of PCC at clinically relevant and lower concentrations in whole blood samples of healthy adults using the NATEM assay. They assumed that heparin-free PCC has no heparin effect and came to the conclusion that the heparin effect was significant in thromboelastometry parameters, at clinically relevant PCC concentrations. Another study by Oda et al. [34] intended to evaluate the effects of amniotic fluid on fibrinolysis and blood coagulation using thromboelastometry NATEM parameters in 21 term-singleton pregnant women with an amniotic fluid infection. ROTEM may demonstrate all hemostatic changes caused by whole blood contamination with amniotic fluid. Based on the results that came from the NATEM and fibrin-based extrinsically activated test by using tissue factors and the platelet inhibitor cytochalasin D (FIBTEM) FIBTEM assays application, such as shortened CT in NATEM and FIBTEM and increased clot firmness parameters A10 and MCF only in NATEM, the authors came to the conclusion that amniotic fluid enhanced the blood coagulation by induced thrombin generation and activated platelet aggregation without changes to fibrin formation and stability.

#### 3.2.2. NATEM Assay Application in Healthy Human Volunteers

There were eight studies regarding the NATEM assay application in a healthy human population (Table 2).

Schneider et al. [31] assessed thrombin generation in 132 healthy adults and its correlation toROTEM parameters. The authors found that the age-related changes in calibrated automated thrombogram (CAT) and in ROTEM variables among adults are not linear. There was a positive correlation between age and maximum clot firmness and alpha angle, but a negative correlation between age and clotting time. There was a significant correlation, although with a moderate slope, but further studies are required. Schöchl et al. [15] conducted an observational study and revealed a similar coagulation profile in thromboelastometric measurements between humans and baboons. The major difference between the species appeared to be a higher resistance of the clot to fibrinolytic breakdown in baboons, as reflected by the NATEM parameters. Furthermore, Spiezia et al. [24] aimed to study the application of whole blood ROTEM between humans and cynomolgus monkeys in order to evaluate the characteristics of clot formation between these two species. ROTEM was found to demonstrate a hypercoagulable profile in monkeys compared to humans, which could consequently lead to a more difficult xenotransplantation in monkeys than in humans.

In addition, Jilma-Stohlawetz et al. [25] aimed to examine the circadian variation and the between-and within- subject variation in ten healthy adults within an 8-week period using the NATEM assay, as well as the influence of elevated body mass index (BMI) and the effect of antithrombin and low molecular weight, on the formation of the clot. They found a high daily variation and a significant impact of high BMI on clotting parameters; however, these findings have a minor clinical relevance in emergency situations. NATEM assay is sensitive to the prothrombotic phenotype in obese individuals, as well as the within one day changes of hemostatic profile of healthy adults which reflect the circadian variability of components involved in hemostasis.

Moreover, Getrajdman et al. [36] aimed to define the normal values for non- activated thromboelastometry parameters among 120 healthy pregnant women, through a prospective observational study. This was the first study reporting the reference ranges for NATEM assay with or without heparinase in non–laboring pregnant women, indicating that NATEM method could be useful in monitoring anticoagulant treatment in pregnant women. Last, Lechien’s et al. [30] conducted an observational study in order to determine the tranexamic acid (TXA) concentration that is required to inhibit 95% (EC95) of tissue-type plasminogen activator (t-PA)-induced fibrinolysis in healthy pregnant and non pregnant women. The outcomes of this study indicate that pregnant women have a higher fibrinolytic potential compared with nonpregnant women.

Furthermore, Sidlik et al. [42] aimed to examine the functional fibrinolytic capacity of newborn’s cord blood using NATEM. Lysis parameters such as lysis index at 30 min (LI30) and time to lysis (TTL), tested with increased t-PA-concentration, were found significantly lower in neonate’s cord blood. This was the first study regarding the functional fibrinolysis in cord blood, and the findings indicated that neonatal fibrinolysis is increased compared to adults. Sulaj et al. [32] reported reference ranges for NATEM parameters in healthy-term neonates using cord blood samples and showed that the gestational age had a positive correlation with lysis index at 30 and 60 min; these results are in accordance with other studies performed in healthy neonates using intrinsically activated test with ellagic acid (INTEM) and extrinsically activated test with tissue factor (EXTEM) assays [43,44]. Moreover, female neonates appear to have a more hypercoagulable profile compared to males, manifested with faster CT and higher clot amplitude at 20 min (A20) and MCF; a finding which can attributed to the increased levels of coagulation factors that females present [45].

#### 3.2.3. Comparison of NATEM Parameters between Healthy Adult Volunteers and Patients

Seven studies comparing healthy adults and patients were identified (Table 3).

Meesters et al. [13] investigated the correlation of NATEM results between healthy adults and patients with Systemic Inflammatory Response Syndrome (SIRS) and found that in citrated blood samples, NATEM results are modified over time and, consequently, a NATEM test should be carried out directly after blood sampling, as opposed to activated ROTEM assays such as INTEM/EXTEM/FIBTEM. Furthermore, Treliński et al. [17] evaluated ROTEM parameters in patients with multiple myeloma (MM) in order to identify those with the highest risk of developing thrombotic events and revealed that a NATEM test could identify a prothrombotic situation in patients with MM. Additionally, Spiezia et al. [20], through a case–control study, assessed the coagulation profile of 30 pregnant women with preeclampsia and 60 healthy pregnant women using NATEM and they found that pregnant women with preeclampsia are in a prothrombotic state not only due to a higher clot stability, but also due to a decrease in fibrinolysis. Livnat et al. [40], in a study enrolling 25 patients with severe FXI deficiency and 16 healthy adult volunteers, used a NATEM assay and found that it was improper to identify patients at increased risk bleeding following a surgical procedure. Lastly, Furukawa et al. [35] evaluated a monitoring protocol using NATEM in patients with hemophilia A (HA) with inhibitors on bypassing treatment, and concluded that NATEM could offer a promising strategy concerning bypassing therapy in HA patients receiving inhibitors. Additionally, Rossetto et al. [14] analyzed the whole blood ROTEM values between cirrhotic and non-cirrhotic patients with portal vein thrombosis (PVT) and a group of healthy volunteers. NATEM and traditional coagulative parameters (i.e., platelet count, prothrombin time/international normalized ratio (PT/INR), activated partial thromboplastin time (aPTT), and fibrinogen) were assessed on blood samples from each group; no significant differences were found between the control group and PVT patients, both with and without cirrhosis. Recently, Aires et al. [41] have evaluated the hemostatic status of COVID-19 patients using ROTEM EXTEM, INTEM, FIBTEM and NATEM assays. The authors evaluated the ROTEM parameters of nonsevere and severe forms of COVID-19 patients compared to healthy controls and reported that the altered NATEM-CT would represent a thromboelastometric parameter useful as a predictor of disease severity.

#### 3.2.4. NATEM Assay Application in Different Clinical Settings

Finally, regarding our fourth group of articles (NATEM assay application in patients) we revealed eight studies (Table 4).

In a large cohort study, Shalaby et al. [19] aimed to assess the coagulation and endothelial damage in the portal vein in 45 cirrhotic patients with portal hypertension by using NATEM method. The indications of damaged glycocalyx, in combination with increased concentrations of endothelial microparticles (MPs), glycosaminoglycans (GAGs), and endotoxemia levels (lipopolysaccharide (LPS)), suggest an important alteration of the endothelium in the portal vein compared to peripheral veins; as a result, this endothelial damage could be indicative of a significant local risk factor in the pathogenesis of portal vein thrombosis.

Brearton et al. [21] aimed to examine the performance of the viscoelastic coagulation monitoring system (VCM) by comparing it to the ROTEM testing device among 86 patients undergoing a planned major abdominal, orthopedic or vascular surgery. They revealed that the VCM system is not only an easy-to-use method, but also capable of making measurements of patients’ coagulation system. A good correlation between the most VCM and NATEM parameters (CT, A10, A20 and MCF) was reported. Moreover, Silverberg’s [22] goal was to compare citrated and fresh whole blood by using a NATEM assay and Sonoclot tests in 38 patients during elective neurosurgery. They showed that the citrated blood induced a hypercoagulative response, as compared to the fresh whole blood parameters, in NATEM clot formation time and α-angle, Sonoclot platelet function and clot rate.

A case report by Durila et al. [26] demonstrated that non-activated thromboelastometry was capable of demonstrating fibrinolysis in a bleeding patient, in contrast to activated assays. In addition, Lancé et al. [28] studied the influence of pneumatic tube system (PTS) transport on ROTEM results and its contribution to investigating the activation evaluated by thrombin generation (TG). They reached the conclusion that, among the ROTEM analysis, NATEM methods are the most sensitive tool to reflect small changes in the coagulation system. Rapid transport in pneumatic systems may influence the reliability of results by platelet activation and/or contact activation, as a NATEM assay revealed it was expressed with a shortened CT, which reflects the initiation of clot formation. Ultimately, Yeom et al. [38] aimed to examine the use of thromboelastometry assays in testing coagulation disorders and its contribution in factor replacement in congenital FVII deficiency, and showed that NATEM analysis indicated a normal coagulation condition in a 22-year-old woman with an FVII:C of 8%; however, she received activated recombinant FVII (rFVIIa), which most probably led to thrombosis of the radial artery. Yada et al. [33] assessed the coagulation status of seven patients with hemophilia A using NATEM. The authors found that there was a significant dose-dependent shortening of CT and CT + CFT times following emicizumab administration in these patients and they concluded that NATEM could be used to evaluate hemostasis in patients with hemophilia A receiving emicizumab. Nevertheless, in a large cohort study by MacDonald et al. [27] of patients with an unclassified bleeding disorder, a NATEM assay was not able to contribute to the classification of hemorrhagic patients. Similarly, in a study by Livnat et al. [40] including 25 patients with severe FXI deficiency and 16 healthy adult volunteers, a NATEM assay was incapable of predicting bleeding diathesis in such patients undergoing a surgical procedure. Additionally, Rossetto et al. [14] analyzed the whole blood ROTEM values between cirrhotic and non-cirrhotic patients with portal vein thrombosis (PVT) and a group of healthy volunteers. No significant differences were found on NATEM parameters and conventional coagulation tests between control group and PVT patients, both with and without cirrhosis.

## 4. Discussion

A NATEM assay provides a comprehensive insight into the hemostatic mechanism and is a highly sensitive method to detect endogenous coagulation activators such as the tissue factor. Although the interest in viscoelastic methods (TEG and ROTEM) has recently grown in the fields of hemostasis and therapeutic transfusion in adults and neonates, there is only limited evidence in the literature regarding the detecting ability of NATEM assays for hemostatic disorders in neonates or adults [46,47,48]. The published data on NATEM are scarce compared to INTEM, EXTEM, and FIBTEM assays. It is noteworthy that use in neonates is still limited due to the lack of established reference values. Additionally, there are no comparative studies demonstrating the superiority of NATEM over EXTEM or INTEM in the prognosis of hemostatic disorders and/or in predicting the occurrence of bleeding events.

A NATEM assay evaluates both the intrinsic and the extrinsic coagulation pathways without any reagents for the activation of coagulation. As opposed to the EXTEM and INTEM assays, which use tissue factor and ellagic acid, respectively, to activate coagulation, NATEM only contains calcium to reverse the action of citrate in the sample tubing. Therefore, since no coagulation activators are required in the NATEM assay, this method could potentially provide a more precise depiction of the in vivo hemostatic mechanism compared to EXTEM and INTEM assays. Finally, NATEM is cheaper than EXTEM and INTEM, since no reagent activator is required. Due to all these advantages of the NATEM assay, this laboratory method is an attractive option for an easy and rapid evaluation of hemostasis, especially in the neonatal population. A disadvantage of the NATEM assay, however, is the longer running time that is required for results compared to the other ROTEM assays.

Although the results of the activated ROTEM assays, such as INTEM, EXTEM and FIBTEM assays, remain unaffected in blood samples stored for up to 2 h, most NATEM parameters, such as CT and CFT, are significantly affected by the time interval between the blood draw and sample analysis [49,50]. It is hypothesized that this is caused by the degradation of citrate in red blood cells and platelets via the citric acid cycle and the subsequent increase in calcium and coagulation reactivation [51,52]. Another possible cause of the storage time impact on NATEM parameters is the activation of the intrinsic coagulation pathway following contact the of coagulation factor XII with kaolin or glass. Factor XIIa leads to the of factor XI and consequent activation of factor IX, in the presence of calcium, resulting in clotting time reduction [53]. The accumulation of microparticles with procoagulant potency from activated cells could also justify alterations in the NATEM parameters after sample storage [54,55]. Therefore, a NATEM assay should be performed in freshly drawn citrated blood in order to prevent bias by different initiation times and be more representative of patients’ hemostatic status [13].

A NATEM assay has been reported as a potential useful laboratory test to evaluate coagulopathy in patients with various diseases such as hemophilia A [33], cirrhosis with non-neoplastic portal vein thrombosis [19], multiple myeloma [17] and preeclampsia during pregnancy [20]. NATEM could also be used to detect changes in the coagulation activity of patients taking anticoagulant medications. Despite the promising results of the NATEM assay in detecting hemostatic disorders, there were some studies in which a NATEM assay was not able to distinguish bleeders and non-bleeders, nor could it predict hemorrhagic risk in patients with severe FXI deficiency undergoing surgery. In addition, in other studies, NATEM could not classify the hemorrhagic risk and identify coagulation disorders [16,29,42]. Recently, a NATEM assay was reported as a useful tool in the differentiation between severe and nonsevere COVID-19 patients [41].

In general, the most frequently used ROTEM assays are EXTEM, INTEM, and FIBTEM. The EXTEM and INTEM assays provide information on the extrinsic and intrinsic coagulation pathway, respectively, as well as on their interaction with platelets, and constitute the initial steps of screening. FIBTEM clot firmness parameters such as A5, A10, and MCF determine the fibrinogen level and fibrin polymerization; in combination with the corresponding EXTEM parameters, they can also delineate the hypofibrinogenemia from isolated thrombocytopenia, both of which contribute as key players to a clot’s rigidity and elastic properties. The FIBTEM parameters are currently used in clinical algorithms and protocols aiming to guide and individualize goal-directed therapy in several clinical settings such as in trauma-induced coagulopathy and in demanding perioperative management. Taking into account both the bleeding and the thrombotic risk, common complications in patients undergoing surgery, the perioperative management of hypofibrinogenemic patients or those receiving anticoagulants is complicated, and an assessment of the patient’s hemostatic status is required. ROTEM and TEG can assist in perioperative coagulation management [48,56,57]. Although EXTEM and INTEM assays are commonly used in algorithms to guide transfusion for the management of severe bleeding, increased bleeding diathesis can be evident despite normal EXTEM or INTEM values. EXTEM/INTEM methods are used in an emergency setting when information on the patient’s hemostatic profile is required promptly. However, it should be noted that the reagents used for in the vitro activation of coagulation in the above methods may provide erroneous results. In fact, although activation of the extrinsic system by tissue factor resembles the natural process, several issues emerge. Different sources of tissue factor and its physiologically varying biologic potency in different lots create variability. Additionally, the extent of the involvement of the contact system also contributes to the variability of results [9,58,59,60]. On the contrary, the NATEM method, in which coagulation activation relies solely on calcium administration, can reveal coagulation disorders that are not detected by EXTEM/INTEM [4,26]. Our systematic review of the literature revealed limited evidence supporting the superiority of NATEM compared to EXTEM/INTEM in the diagnosis and prognosis of hemostatic disorders. However, NATEM seems to be a sensitive method for the detection of hyperfibrinolysis [26,42] and may have an advantage in the diagnosis of those hemostatic disorders in which dysfunctional fibrinolysis is mainly involved. In vitro studies have demonstrated that the NATEM assay could be a useful method to monitor the degree of endothelial damage and has a high sensitivity to detect changes in the coagulation status of patients following PCC administration [16,19]. Moreover, it was shown that a NATEM assay can provide more detailed information than the other ROTEM assays about the coagulation status of patients with hemophilia A who receive prophylactic treatment with emicizumab [33].

## 5. Conclusions

NATEM has high sensitivity to detect hypo- or hypercoagulability and might provide a detailed insight onto the whole hemostatic process from clot formation to clot breakdown. It could be a useful method to monitor the coagulation status not only of adults, but children and neonates as well, while it might also be used to guide hemostatic therapy and predict certain clinical outcomes such as mortality/morbidity in those patients. However, the reported data regarding the diagnostic/predictive value of a NATEM assay for coagulopathies and its efficacy to guide hemostatic therapy are highly varying among the published studies. There is a need to clearly define its reference range and thresholds to diagnose hemostasis disorders, predict increased bleeding or mortality rate, and guide the hemostatic resuscitation in a neonatal or adult population.

## Figures and Tables

**Table 1:** Non-activated rotational thromboelastometric (NATEM) assay in experimental studies.

Author, Publication Year, Country	Study Design, Centers, Time Period	Study Population	Objective	Results/Conclusions
Oda, 2018 Japan [34]	In vitro experimental	21 (pregnant women with amniotic fluid infection)	NATEM in pregnant women with amniotic fluid infection	Based on the results that came from the NATEM and FIBTEM assay application, the authors came to the conclusion that amniotic fluid enhanced blood coagulation, by induced thrombin generation and activated platelet aggregation, without changes to fibrin formation and stability.
Bar, 2017 USA [37]	Single center, prospective, experimental pilot	8 (adult patients)	NATEM in patients receiving Rivaroxaban	The NATEM test could detect changes in coagulation parameters induced by hemostatic agents in patients receiving Rivaroxaban/chitosan or kaolin-based hemostatic agents may be effective in improving these patients’ hemostasis profile.
Bagge, 2016 Sweden [23]	Single center, prospective, pilot, experimental	35 (healthy adults)	Effects of naturopathic medicines on NATEM	Standard intake of 1260 mg Ω-3 of polyunsaturated fatty acids (fish oil) daily may decrease platelet aggregation and clot formation.
Elvstam, 2016 Sweden [18]	Single center, experimental	10 (healthy adults)	NATEM and dilutional coagulopathy	The NATEM assay has been described as the most sensitive protocol for detecting endogenous tissue factors, and it may be preferred for monitoring the Prothrombin complex concentrate (PCC) effects of dilutional coagulopathy.
Zipperle, 2013 Austria [16]	In vitro experimental	Healthy adult volunteers	NATEM and adherent endothelial cells	Adherent endothelial cells participate in the process of hemostasis and fibrinolysis and could be incorporated into ROTEM assays via coated microbeads. In the NATEM assay, only a significant reduction in CT value was observed in the presence of unstimulated Endothelial cells, while on the other hand, CFT, a-angle, A30 and MCF values did not show any difference.
Scharbert, 2012 Austria [29]	Experimental	10 (healthy adults)	NATEM and heparin-induced effects of prothrombin complex	Heparin effect was significant in thromboelastometry at clinically relevant PCC concentrations.
Parsi, 2011 Australia [39]	Single center, experimental	Healthy adults	NATEM and sclerosants	Detergent sclerosants indicated a trimodal reaction on clot formation, initiating strong clots at low concentrations, weak clots at midrange concentrations and preventing clot formation at higher concentrations.

Abbreviations: non-activated rotational thromboelastometric (NATEM); prothrombin complex concentrate (PCC), clotting time (CT); clot formation time (CFT); maximum clot firmness (MCF); amplitude at 10, 20 min (A10,20).

**Table 2:** Non-activated rotational thromboelastometric (NATEM) assay in healthy human volunteers.

Author, Publication Year, Country	Study Design, Centers, Time Period	Study Population	Objective	Results/Conclusions
Sulaj, 2021, Greece [32]	Single center, prospective, observational, March 2021–November 2021	189 healthy-term neonates	NATEM parameters in cord blood samples of healthy-term neonates.	Demonstrate reference ranges for healthy-term neonates in NATEM assay
Getrajdman, 2021 USA [36]	Single center, prospective, observational, July 2019–February 2021	120 (healthy pregnant women)	NATEM in healthy pregnant women	NATEM method could be useful in monitoring anticoagulant treatment in pregnant women
Lechien, 2021 Germany [30]	Observational	40 (30 full term pregnant women vs. 10 healthy non pregnant women)	NATEM and tranexamic acid in Postpartum hemorrhage	Pregnant women have a higher fibrinolytic potential compared with nonpregnant women
Jilma-Stohlawetz, 2017 Austria [25]	Single center, observational	42 (healthy adults) −10 healthy, BMI 23 ± 3 kg/m^2^ −17 healthy, BMI > 30 kg/m^2^ −15 healthy (12 Male/3 Female)	Circadian variation, BMI evaluation and effect of LMWH and recombinant antithrombin on coagulation and fibrinolysis	A high daily fluctuation and an influence of high BMI on clotting parameters was found. NATEM assay is sensitive to the prothrombotic phenotype in obese individuals, as well as the within one day changes of hemostatic profile of healthy adults which reflect the circadian variability of components involved in hemostasis.
Sidlik, 2016 Israel [42]	Single center, prospective, cohort	124 (101 neonates and 23 healthy adults)	NATEM in neonatal population	Lysis parameters such as lysis index at 30 min (LI30) and time to lysis (TTL), tested with increased tPA-concentration, were found significantly lower in neonate’s cord blood fibrinolysis was more rapid in the newborns
Schneider, 2015 Germany [31]	Single center, observational	132 (healthy adults)	NATEM and healthy adults	The age-related changes in calibrated automated thrombogram (CAT) and in ROTEM variables among adults are not linear
Schöchl, 2012 Austria/Germany [15]	Observational	46 (25 baboons and 21 human volunteers)	NATEM between baboons and humans	Similarities in thromboelastometric measurements between humans and baboons/a higher resistance of the baboon clot to fibrinolytic breakdown measured in NATEM
Spiezia, 2010 Italy [24]	Observational	93 (43 monkeys vs. 50 healthy adults)	Reference values for NATEM in monkeys	A hypercoagulable profile in monkeys as compared to humans/probably more difficult is xenotransplantation in monkeys than in humans

Abbreviations: Non-activated rotational thromboelastometric (NATEM); Body mass index (BMI); Low-molecular-weight heparins (LMWHs); Clotting Time (CT); Clot Formation Time (CFT).

**Table 3:** Comparison of NATEM parameters between healthy adult volunteers and patients.

Author, Publication Year, Country	Study Design, Centers, Time Period	Study Population	Objective	Results/Conclusions
Rossetto, 2013 Italy [14]	Single center, observational, January 2010–September 2012	63 (49 cirrhotic or non-cirrhotic patients with or without PVT and 14 healthy adults)	NATEM in patients with PVT cirrhotic or non-cirrhotic	There were no significant differences in NATEM assays and in traditional coagulative parameters between the two groups.
Treliński,2014 Poland [17]	Single center, observational	46 (26 patients with MM and 20 healthy adults)	NATEM and patients with multiple myeloma	A NATEM test could contribute to identify a prothrombotic situation in patients with MM.
Spiezia, 2015 Italy [20]	Single center, case–control, December 2013–September 2014	90 (30 pregnant with preeclampsia vs. 60 pregnant healthy women)	NATEM in pregnant women with preeclampsia	A prothrombotic state in pregnant women with preeclampsia was found compared to healthy pregnant women.
Meesters, 2015 Netherlands/Germany [13]	Multicenter, cohort	44 (14 healthy adults and 30 patients admitted to ICU)	NATEM in citrate stored blood	A NATEM test should be carried out directly after blood sampling.
Livnat, 2015 Russia [40]	Cohort, January 2013–February 2014	41 (25 patients vs. 16 healthy adults)	NATEM and patients with FXI deficiency	A NATEM assay was incapable of predicting bleeding predisposition of the patients.
Furukawa, 2015 Japan [35]	Single center, cohort	28 (8 patients vs. 20 healthy adults)	NATEM and hemophilia A	A NATEM could offer a promising strategy concerning bypassing therapy in HA patients receiving inhibitors.
Aires, 2022 Brazil [41]	Single center, observational, 1 August and 30 September 2020	61 (41 patients with COVID-19 and 20 healthy adults)	NATEM and patients with COVID-19	The altered NATEM–CT would represent a thromboelastometric parameter useful as a predictor of disease severity.

Abbreviations: non-activated rotational thromboelastometric (NATEM); multiple myeloma (MM); portal vein thrombosis (PVT); clotting time (CT).

**Table 4:** NATEM assay application in different clinical settings.

Author, Publication year, Country	Study Design, Centers, Time Period	Study Population	Objective	Results/Conclusions
Yeom, 2021 USA [38]	Case report	1 (patient female with severe FVII deficiency)	NATEM and patient with FVII deficiency	NATEM analysis indicated a normal coagulation condition.
Shalaby, 2020 Italy [19]	Single center, cohort	45 (cirrhotic patients with PVT)	NATEM and PVT in cirrhotic patients	NATEM assay highlighted the presence of a heparin-like effect in portal blood reflecting the endothelial dysfunction in the portal vein.
MacDonald, 2019 UK [27]	Large cohort, Single center, 2001–October 2018	124 (patients with unclassified bleeding disorder)	NATEM and patients with unclassified bleeding disorder	NATEM assay was not able to contribute to the classification of hemorrhagic patients.
Yada, 2019 Japan [33]	Single center, cohort, May 2013–February 2016	7 (adult patients with HA receiving emicizumab)	NATEM in patients with hemophilia A who received emicizumab	NATEM could be useful for the evaluation of hemostasis status in patients with Hemophilia A receiving emicizumab.
Brearton, 2019 UK [21]	Single center, observational	86 (patients undergoing abdominal, orthopedic or vascular surgery)	Comparison of NATEM and VCT	VCM system is not only an easy-to-use method but also capable of making measurements of patients’ coagulation system. There was reported a good correlation between the most VCM and NATEM parameters (CT, A10, A20 and MCF).
Silverberg, 2017 Sweden/Austria [22]	Prospective, unblinded observational cohort, September–December 2015	38 (patients undergoing elective brain tumor resection/biopsy)	NATEM and comparison of citrated and fresh whole blood during elective neurosurgery	The citrated blood indicated a hypercoagulative response as compared to the fresh whole blood parameters.
Durila, 2016 Prague [26]	Case report	1 (patient with ards and infection)	NATEM and detection of bleeding disorder in a patient with aRSD	NATEM was capable of distinguishing fibrinolysis in a bleeding patient, in contrast to activated assays.
Lancé, 2012 Netherlands [28]	Observational	44 (patients scheduled for elective cardiothoracic surgery)	NATEM and pneumatic tube system (PTS)	Among the ROTEM analysis, NATEM methods is the most sensitive tool to reflect small changes in the coagulation system. The rapid transport in pneumatic systems may influence reliability of results by platelet activation and/or contact activation as it expressed with a shortened CT revealed in NATEM assay, which reflects the initiation of clot forming.

Abbreviations: non-activated rotational thromboelastometric (NATEM); multiple myeloma (MM); portal vein thrombosis (PVT); viscoelastic coagulation test (VCT); acute respiratory distress syndrome (Ards); pneumatic tube system (PTS).
